# Pulse modulation in En-Bloc HoLEP: does it really matter? A propensity score matched analysis

**DOI:** 10.1007/s00345-024-04830-8

**Published:** 2024-03-14

**Authors:** Friedrich Otto Hartung, Luisa Egen, Britta Gruene, Maren Juliane Wenk, Karl-Friedrich Kowalewski, Paul Patroi, Marie-Claire Rassweiler-Seyfried, Maurice Stephan Michel, Jonas Herrmann

**Affiliations:** Department of Urology and Urologic Surgery, University Medical Center Mannheim, University of Heidelberg, Theodor-Kutzer-Ufer 1-3, 68167 Mannheim, Germany

**Keywords:** MoLEP, HoLEP, Holmium, MOSES, Endoscopic enucleation of the prostate, Prostatic hyperplasia, BPH

## Abstract

**Introduction:**

Holmium laser enucleation of the prostate (HoLEP) is an established option in the surgical treatment of benign prostatic hyperplasia. Pulse modulation, such as MOSES® technology, has recently been introduced and may offer potential advantages in HoLEP.

**Methods:**

Perioperative data from 117 patients who underwent MOSES® laser enucleation of the prostate (MoLEP) were collected. Propensity score matching using prostate volume, age, body mass index (BMI), and anticoagulant intake was performed using a database of 237 patients treated with HoLEP. In total, 234 patients were included in the analysis.

**Results:**

Prostate volume (104 vs. 102 ml), age (70 vs. 71 years), BMI (27 vs. 27), and anticoagulant intake (34 vs. 35%) did not differ significantly between the groups. There were no significant differences in operation time (61.5 vs. 58.1 min, p = 0.42), enucleation efficiency (2.5 vs. 2.6 g/min, p = 0.74), hemostasis time (7.8 vs. 8 min, p = 0.75) and hemoglobin drop (0.9 vs. 0.7 mg/dl, p = 0.48). The complication rates were low in both groups (16.2% for HoLEP and 17.1% for MoLEP). No differences were noted in the Clavien-Dindo Classification (p = 0.63) and the Comprehensive Complication Index (p = 0.24). The rate of complications > CDC IIIa was 0.9% for HoLEP (endoscopic coagulation) and 1.7% for MoLEP (2 cases of endoscopic coagulation). No transfusions were administered.

**Conclusion:**

Overall, the enucleation efficiency was high in both groups and the procedure time was short. HoLEP is an efficient and safe treatment option in experienced hands, regardless of the use of pulse modulation technology.

## Introduction

Owing to its efficacy, reproducibility, safety, and universal applicability regardless of prostate volume, endoscopic enucleation of the prostate (EEP) is emerging as the new gold standard for surgical treatment of lower urinary tract symptoms (LUTS) caused by benign prostatic hyperplasia (BPH) [1, 2]. Among the numerous energy sources available for EEP, Holmium YAG (Ho-YAG) lasers are the most widely used [3]. Although the efficacy and safety of different energy sources appear to be similar [3], Ho-YAG has several unique features that makes it particularly suitable for anatomical enucleation [4]. One distinguishing feature is the production of a vapor bubble with each laser pulse. The eruption of this vapor bubble, the size of which is determined by the energy of the laser pulse, generates a mechanical force that is directed not only in the direction of the laser beam but also laterally. This characteristic enables mechanical separation of the dissection plane without the use of force with the tip of the endoscope.

Lumenis introduced MOSES® Technology, a pulse modulation technology for laser surgery, which makes use of the Moses effect, as described in 1986 by Isner et al. [5, 6]. It was designed primarily for stone treatment, where it offers numerous substantial advantages over the standard pulse settings. In theory, the pulse is broken into two peaks, where the bubble generated by the first peak separates the water, allowing the second peak to travel through the first bubble, changing the structure of the bubble and facilitating energy transmission to the target tissue [7]. In lithotripsy, this leads to relevant improvements such as decreased retropulsion, decreased fiber vibration, and increased ablation efficacy [8]. Recently, several prospective and retrospective studies have investigated the application of pulse modulation in HoLEP with heterogeneous results [9–12].

This study aimed to examine the impact of MOSES® technology in HoLEP on perioperative outcomes and complications in a large real-life cohort of a single high-volume surgeon.

## Methods

### Study design

Data was retrieved from a database at the Department of Urology and Urological Surgery at the University Medical Center Mannheim, Heidelberg University (institutional review board approval 2021-882).

We recruited 117 patients who were treated for BPH by MoLEP from January 2021, the year MoLEP/HoLEP was first introduced in our center, to March 2023 and evaluated the following parameters: age at surgery, body mass index (BMI), prostate volume, and anticoagulant intake. The anticoagulants included coumarins, indandiones, factor Xa inhibitors, heparins, thrombin inhibitors and inhibitors of platelet aggregation. Based on these parameters, propensity score matching was performed using a database of 237 patients treated with HoLEP without pulse modulation between 2021 and March 2023. Among the matched patients, intra- and postoperative parameters such as operation time (OT), enucleation weight (EW), enucleation time (ET), hemostasis time (HT), morcellation time, lasing time, enucleation efficiency (EF = EW/ET), postoperative catheterization time, length of hospital stay, hemoglobin drop, transfusion rate, postoperative urinary retention, reintervention rate (surgical, endoscopic or radiological in terms of urinary catheter insertion under radiological fluoroscopy), Clavien-Dindo Classification (CDC) [13] and the Combined Complication Index (CCI) [14] were evaluated. Hemoglobin drop was defined as the difference between the hemoglobin concentration preoperatively and one day after surgery. The transfusion rate was documented for the length of the hospital stay. To record and classify postoperative complications, the CDC and CCI were calculated for each patient.

### Procedures

All procedures were performed in consecutive order by one experienced endoscopic surgeon who previously performed > 100 endoscopic enucleations with both thulium and holmium lasers. A Lumenis Pulse 120H Holmium-Lasersystem with MOSES® 2.0 technology (Boston Scientific Corporation, Massachusetts, USA) was used. Either MOSES® 550 single use fiber for MoLEP or SlimLine™ SIS 550 single use or reusable fiber for HoLEP were used. Pulse modulation was used depending on fiber availability and surgeon´s discretion. All operations were performed using the En-Bloc technique described by Saitta et al. [15]. Laser settings were set to 2 Joule and 50 Hertz for the enucleation in both HoLEP and MoLEP. Coagulation settings were set at 1 Joule and 40 Hz long pulse. A 26 French continuous flow laser resectoscope with a Kuntz working element and a Wolf Piranha Morcellator were used in all cases (Richard Wolf GmbH, Knittlingen, Germany). Bipolar electrocautery was used at the end of the procedure to ensure optimal hemostasis.

### Statistical analysis

Propensity score matching was performed using the R package “MatchIt” [16]. A propensity score was allocated to each patient using the nearest neighbor method and age, BMI, prostate volume, and the intake of anticoagulants as covariates. Each treated patient (MoLEP) was matched in a 1:1 ratio to one patient from the control group (HoLEP) based on the closest value of the propensity score. All parameters are presented as mean ± standard deviation (SD) for continuous data. For binary parameters, absolute and relative frequencies are given. Group comparisons were performed using the unpaired t-test for continuous and the Chi^2^-test for binary outcomes. A p-value of p ≤ 0.05 was considered significant. All analyses were performed using R, version 4.3.0 (R-Studio, R Foundation for Statistical Computing, Vienna, Austria) [17].

## Results

**Fig. 1:**
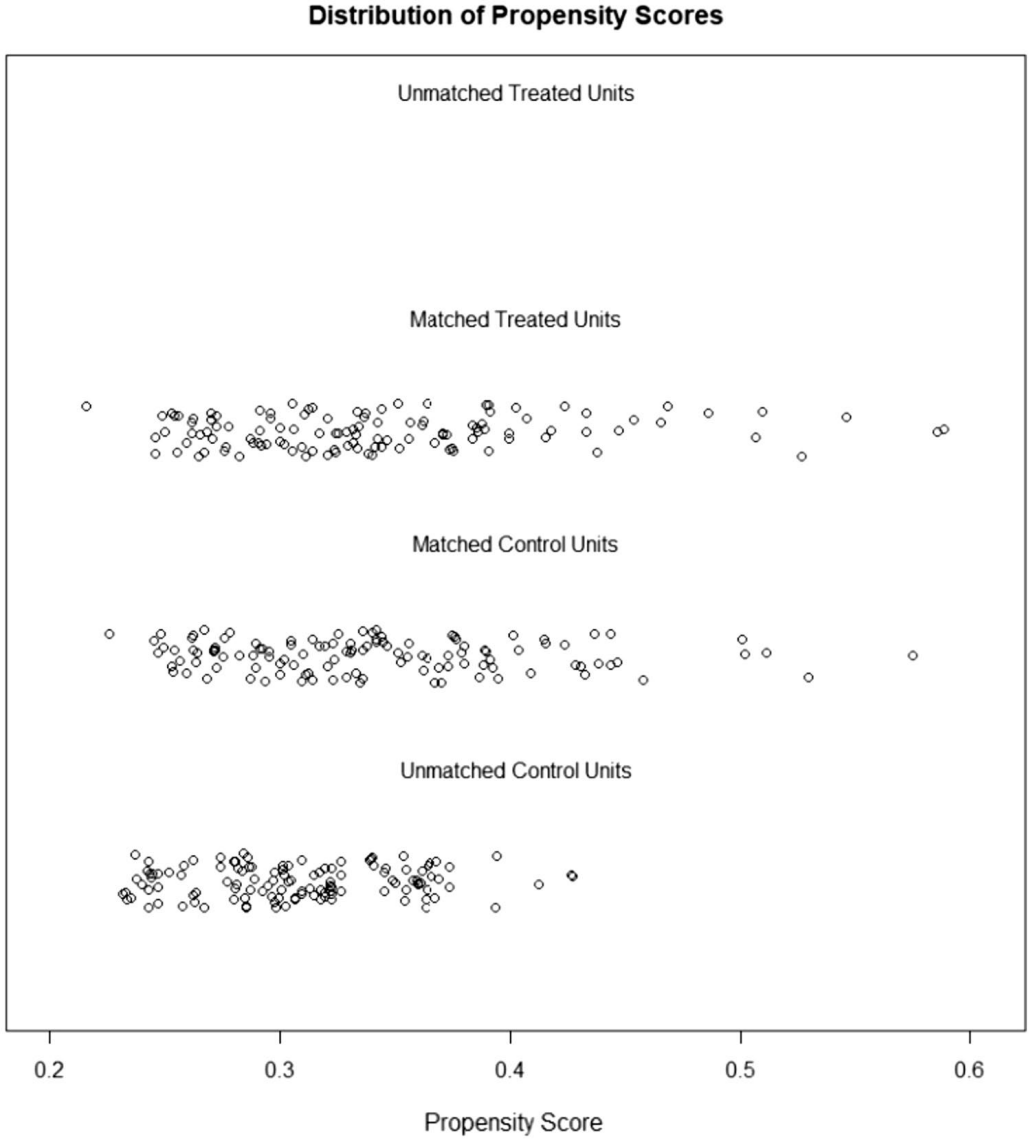
Distribution of propensity scores

117 MoLEP patients were matched to 117 HoLEP patients, leaving 234 patients for the final analysis (Fig. 1).

After matching, the groups were balanced in terms of baseline characteristics (Table 1).

**Table 1:** Overview of baseline data before and after propensity score matching

	Pre-matching			Post-matching		
	HoLEP (n = 237)	MoLEP (n = 117)	p-value	HoLEP (n = 117)	MoLEP (n = 117)	p-value
BMI, kg/m^2^ Mean ± SD	27 ± 4	27 ± 5	0.58	27 ± 4	27 ± 5	0.91
Age, years Mean ± SD	71 ± 8	70 ± 9	0.87	71 ± 8	70 ± 9	0.83
Prostate volume, ml Mean ± SD	91 ± 45	104 ± 59	**0.02**	102 ± 53	104 ± 59	0.78
Intake of anticoagulants Absolute (%)	100 (42)	41 (35)	0.24	42 (36)	41 (35)	1

n = number of patients, SD = standard deviation, % = percentage, HoLEP = holmium laser enucleation of the prostate, MoLEP = MOSES® laser enucleation of the prostate, BMI = body mass index

### Perioperative parameters and complications

Table 2 shows a comparison of the perioperative parameters and complications between the two groups. For the overall OT, the EF, and the HT, no significant difference was seen. In terms of complications, no significant differences were observed regarding postoperative urinary retention, transfusion rate, and reintervention rate. These were also included in the CDC and CCI.

**Table 2:** Perioperative parameters and complications

	HoLEP (n = 117)	MoLEP (n = 117)	p-value
Mean operation time, min Mean ± SD	61.5 ± 34.2	58.1 ± 30.8	0.42
Enucleation weight, g Mean ± SD	73.3 ± 53.5	70.4 ± 51.7	0.67
Enucleation time, min Mean ± SD	29.3 ± 15.9	27.4 ± 16.3	0.37
Hemostasis time, min Mean ± SD	7.8 ± 4.6	8 ± 4.9	0.75
Morcellation time, min Mean ± SD	17.3 ± 17.4	15.4 ± 11.2	0.31
Lasing time, min Mean ± SD	20.9 ± 19.5	19.1 ± 9	0.37
Enucleation efficiency, g/min Mean ± SD	2.5 ± 1.3	2.6 ± 1.2	0.74
Postoperative catheterization time, days Mean ± SD	1.9 ± 0.8	1.9 ± 1	0.89
Mean LoS, days Mean ± SD	3.7 ± 1.3	3.7 ± 1.7	0.79
Hemoglobin drop, g/dl Mean ± SD	0.9 ± 1.1	0.7 ± 2.1	0.48
Postoperative urinary retention Absolute (%)	7 (6%)	6 (5.1%)	1
Transfusion rate Absolute (%)	0 (0%)	0 (0%)	n.s
Reintervention rate Absolute (%)	1 (0.9%)	2 (1.7%)	0.614
*CDC*			0.63
Grade 0	98 (83.8%)	97 (82.9%)	
Grade I	9 (7.7%)	7 (6%)	
Grade II	9 (7.7%)	9 (7.7%)	
Grade IIIa	0 (0%)	2 (1.7%)	
Grade IIIb	1 (0.9%)	2 (1.7%)	
CCI Mean ± SD	15.79 ± 7.48	19.21 ± 9.58	0.24

n = number of patients, SD = standard deviation, % = percentage, HoLEP = holmium laser enucleation of the prostate, MoLEP = MOSES® laser enucleation of the prostate, LoS = length of hospital stay, CDC = Clavien-Dindo Classification, CCI = Combined Complication Index, n.s. = not specified.

**Fig. 2:**
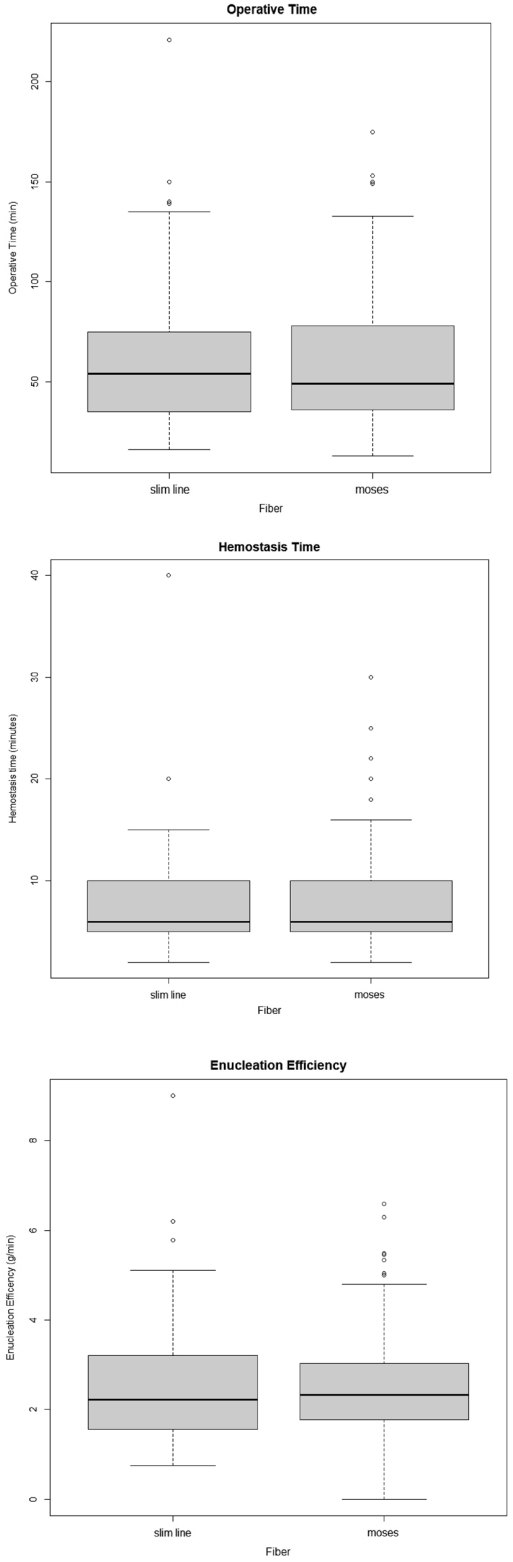
Boxplots

Boxplots for overall OT, HT and EF are shown in Fig. 2

## Discussion

In our study comparing HoLEP and MoLEP, we could not identify any significant differences in perioperative results or postoperative complications. The overall EF was relatively high in both groups. The overall rate of complications was low, and very low for relevant complications greater than CDC grade IIIa.

In MoLEP, pulse modulation alters the shape and properties of the vapor bubble, resulting in several differences: a larger protortion of photothermic energy reaches the target tissue, resulting in a more efficient tissue vaporization and first pass hemostasis [11]. Potential advantages include decreased fiber burnback and vibration as the bubble colapses further away from the tip of the fiber [18]. The altered shape of the bubble results in a decreased amount of mechanical energy directed laterally; however, decreasing the laterally directed mechanical effect unique to Ho-YAG. Therefore, the alteration of the pulse does not come without constraints: the improved cutting and coagulation capabilities are offset by decreased mechanical energy.

With regard to complications, this series adds to the growing body of evidence that HoLEP is extremely safe in experienced hands [19]. Very few significant complications were observed. Bleeding that required intervention in the form of transurethral coagulation was seen in 3 patients (< 1,5%). No transfusions were administered. Two patients had difficulty in recatheterization after urinary retention due to blood clots in the prostatic fossa, which was resolved by flexible cystoscopy. It is not uncommon that catheterisation is challenging after HoLEP due to the steep angle at the level of the bladder neck. While there is nothing wrong with using a flexible cystoscope to overcome the bladder neck, the problem can usually be resolved by the use of a dufour tip catheter with digital rectal guidance. Unsurprisingly, there were no significant differences in complications recorded by both CDC and CCI.

Taking a look at the available studies comparing HoLEP to MoLEP, several things become evident: The results of the different series are highly variable, regardless whether MoLEP or HoLEP was used [20, 21]. The mean EF, which is a suitable parameter to describe the amount of enucleated adenoma per unit time, ranged from 1 to 4.2 g/min between studies [9–12]. Surgical experience was sufficiently high in all series.

Secondly, the one series that used the En-Bloc approach outperformed all other series in terms of EF, HT, and total OT [10] independently whether pulse modulation was used or not. This begs the question of whether the technique applied may not have a greater impact on OT than the energy source used.

Thirdly, the effect of MOSES® technology on surgical results varies between different studies. In the series by Kavrousi et al. [12], EF was equal between the groups. However, there was a significant difference in HT, which ranged from 18 min in the MoLEP group to 29 min in the HoLEP group. Interestingly, HT appeard to be exceedingly long compared to all other series. In the study by Socarrás et al. [10], EF efficiency was significantly better in the MoLEP group. In the study by Nottingham et al., a very slight advantage in enucleation time (45.9 vs. 47.1 min) and hemostasis time (8.1 vs. 10.6 min) could be registered for the MoLEP cohort. In a meta-analyis by Ramadhani et al. [20], the mean difference in OT was not statistically significant. The mean differences in HT and ET were 3.7 and 3.0 min, in favor of MoLEP. It is questionable, however, if these minor differences are clinically relevant. The meta-analysis by Gauhar et al. [21] did show a more pronounced advantage for MoLEP in terms of operation time and hemaostasis time but only included 3–4 Studies in their analysis.

The HT in our cohort was shorter than that in most other studies, probably resulting from hybrid hemostasis with laser coagulation throughout the procedure and bipolar coagulation at the end of the procedure.

Reasons why we did not see a measurable difference in perioperative outcomes may include the following: Since we exclusively used the En-Bloc technique, there were fewer extraanatomical steps in the procedure, and the 5, 7, and 12 o’clock incisions performed in three-lobe technique are probably faster with a modulated pulse. Secondly, most of the studies investigating HoLEP vs. MoLEP performed hemostasis by laser only, rather than the hybrid technique that we applied. This hybrid hemostasis is very common in Germany, although it is less common in the USA and the UK, where most comparative studies of MoLEP and HoLEP were performed. Lastly, as with any surgery, there are surgeon specific differences in the way the procedure is performed. In our experience, a strictly anatomical approach is the most important factor in having an efficient procedure with minimal blood loss and excellent functional results.

This study has limitations due to its unicentric location and as it was not randomized. We used propensity score matching to minimize the risk risk of selection bias. Furthermore, the interventions were all performed by one surgeon with high experience; therefore, the results of this trial may not be generalizable to all surgeons. Although the steep learning curve during the first 100 procedures had already been passed by the surgeon in our series, a longterm learning may still have taken place. Moreover, due to the low overall rate of complications, this study is not powered to detect slight differences in the incidence of complications or other endpoints.

## Conclusions

In this study, no significant differences were observed in perioperative parameters, such as operation time, ET, HT, or blood loss. Furthermore, there were no relevant differences in the complication rates assessed by CDC or CCI criteria. Overall, EF was high and OT was brief in both groups, confirming that HoLEP is a safe and efficient option in experienced hands, regardless if pulse modulation is used or not. The decision to employ pulse modulation technology should be based on the surgeon’s personal preference and availability. To be considered a “gamechanger”, a technological innovation must fundamentally transform a procedure. This, unfortunately, is not the case with pulse modulation technology in HoLEP.

## Data Availability

All data generated or analyzed during this study are included in this article. Further enquiries can be directed to the corresponding author.
